# Trends in the Timeliness of Spinal Muscular Atrophy Detection in US Infants, 2016–2023

**DOI:** 10.3390/ijns12010009

**Published:** 2026-02-18

**Authors:** Scott D. Grosse, Kai Hong, Golriz K. Yazdanpanah, Ashley Nash, Amy Gaviglio, Marcus Gaffney, Kendra A. K. Lawrence, Jennifer M. Kwon

**Affiliations:** 1 Division of Laboratory Sciences, Newborn Screening and Molecular Biology Branch, Centers for Disease Control and Prevention, Atlanta, GA 30341, USA; 2 Department of Pediatrics, University of Minnesota Medical School, Minneapolis, MN 55545, USA; 3 Office of Policy, Performance, and Evaluation, Centers for Disease Control and Prevention, Atlanta, GA 30333, USA; 4 Association of Public Health Laboratories, Silver Spring, MD 20814, USA; 5 Department of Pharmacotherapy, College of Pharmacy, University of Utah, Salt Lake City, UT 84112, USA; 6 Division of Pediatric Neurology, Department of Neurology, University of Wisconsin-Madison School of Medicine and Public Health, Madison, WI 53792, USA

**Keywords:** newborn screening implementation, spinal muscular atrophy, timeliness, evaluation, administrative data

## Abstract

Screening for spinal muscular atrophy (SMA) was adopted by all US state newborn screening programs between 2018 and 2024; by the end of 2022, 48 states were screening for SMA. We assessed trends in health insurance records of SMA diagnoses to quantify improvements in the timeliness of SMA identification following the adoption of screening. We used nationally representative Medicaid claims data for approximately half of US births covered by public insurance and a convenience sample of employer-sponsored health plans. We analyzed records for birth cohorts with at least 1 full year of follow-up (i.e., through the end of the following calendar year). For 2017 births, 1.3 per 100,000 infants had SMA codes first recorded by 1 month of age; this increased to 6.6 per 100,000 among publicly insured newborns born in 2022. The rollout of SMA newborn screening across US states was also followed by improvements in the timely detection of SMA. The proportion of infants with SMA detected by 1 month increased from 18% in 2017 to 61% in 2021 and is projected to reach 75% in 2022. Growth in timely detection was even greater in the employer-insured sample. Timely diagnosis of SMA can enable the initiation of treatment prior to the irreversible loss of motor function.

## 1. Introduction

Over the past 25 years, implementation of newborn bloodspot screening (NBS) methods such as tandem mass spectrometry and the expansion of screening panels to include additional disorders have led to the timely identification and treatment of many thousands of newborns each year and have been highlighted as one of the leading public health successes of the early 21st century [1,2]. The screening pathway includes blood collection via a heel prick onto a blood spot specimen card, transport of the specimen to a laboratory, testing, reporting of results, diagnostic/confirmatory testing, and referral to a provider for treatment once a child is diagnosed. Because delays in this time-sensitive pathway can have adverse consequences for affected newborns, the United States (US) government has set a goal that presumptive positive NBS results for time-critical conditions, defined as conditions that may manifest with acute symptoms in the first days of life and require immediate treatment to reduce the risk of morbidity and mortality, be communicated to the newborn’s healthcare provider no later than 5 days of life [3,4].

One of these time-critical conditions is spinal muscular atrophy (SMA), a genetic disease affecting roughly 1 in 15,000 to 1 in 10,000 children that is caused by mutations in both copies of the survival motor neuron 1 (*SMN1*) gene. As an autosomal recessive disorder, SMA occurs on average in one-fourth of children born to a carrier couple; consequently, a minority of families may have more than one affected child. Reduced production of the SMN protein leads to progressive loss of motor neurons in the spinal cord. SMA type I generally results in death by the age of 2 years in the absence of treatment [5]. The number of copies of the *SMN2* modifier gene, which produces small amounts of functional SMN protein, predicts disease phenotype [6].

Disease-modifying therapies introduced in the past decade include an intrathecal antisense oligonucleotide treatment, nusinersen (Spinraza^®^, Biogen, Cambridge, MA, USA), which was approved by the US Food and Drug Administration (FDA) in 2016. Two other medications were subsequently approved. An intravenous gene therapy using an adeno-associated virus serotype 9 (AAV9) vector, onasemnogene abeparvovec or OA (Zolgensma^®^, Novartis Gene Therapies, Bannockburn, IL, USA), was approved by the FDA in 2019 for children < 2 years of age, and an oral antisense oligonucleotide, risdiplam (Evrysdi^®^, Genentech, South San Francisco, CA, USA), was approved by the FDA in 2020 for US patients of all ages [7]. NBS for SMA was added to the US Recommended Uniform Screening Panel (RUSP) in 2018 on the basis of preliminary evidence of short-term improvements in outcomes with early initiation of treatment (within 6 weeks of birth) with nusinersen [8]. Subsequently, published evidence demonstrated that nusinersen administered to infants significantly improved survival and motor function in infants with 2 or 3 *SMN2* copies [7,9,10]. Immediate treatment for infants with 4 *SMN2* copies has also been recommended by a working group of clinicians and geneticists convened by Cure SMA [11].

Screening for SMA is conducted by amplification of DNA using real-time polymerase chain reaction (RT-PCR) assays to identify individuals with homozygous loss of *SMN1* exon 7, which accounts for roughly 95% of SMA cases [12]. In most US programs, the SMA assay is multiplexed with a PCR-based assay used to screen for SCID and T-cell lymphopenias. The ability to conduct multiplex screening for SMA with SCID, for which all states were screening by 2018, reduced the need for additional instrumentation, reagents, and staff time and enabled the rapid adoption of SMA screening by US NBS programs [13,14]. The multiplexing of SMA and SCID testing has also been reported to be more cost-effective [15].

One US state, Utah, began screening all newborns for SMA in January 2018 [16]. Almost all states had begun screening for SMA by the end of 2022, with the last state beginning screening in early 2024 (Table 1). The pace of adoption of SMA following its addition to the RUSP was rapid relative to that of other conditions added to the RUSP [14,17]. Implementation of SMA NBS in other countries has also grown rapidly since 2021 [18,19].

Based on pooled NBS program data from 30 states for infants born during 2018–2022, 425 (1 in 14,694 births, or 6.8 per 100,000 births) had a confirmed SMA diagnosis [20]. Another analysis of data pooled from 22 states reported a birth prevalence of 1 in 13,862 births, or 7.2 per 100,000 births [21].

## 2. Materials and Methods

We accessed two independent proprietary healthcare administrative databases: Merative™ (Ann Arbor, MI, USA) MarketScan^®^ Commercial data and Centers for Medicare & Medicaid Services (CMS) Medicaid data. Both are closed payer claims databases that record all healthcare encounters billed to participating payers for individuals enrolled in participating plans during the specified time periods. They include records for outpatient and inpatient services and filled outpatient pharmacy prescriptions, with associated diagnosis and procedure codes. Encounters not billed to data contributors are not included (see the Limitations section in the Discussion). Available demographic information for both databases includes age (in years) and sex.

The MarketScan Commercial databases contain person-level, deidentified administrative claims and encounters for privately insured employees and covered family members enrolled in participating employer-sponsored health insurance plans throughout the US. More than 250 self-insured employers and other health plans contributed data for approximately 65 million unique enrollees from 2017 to 2024. Annual numbers of enrollees included in the MarketScan Commercial data decreased over time as a result of changes in contracting arrangements. The Centers for Disease Control and Prevention (CDC) licenses the MarketScan research databases and software for approved public health activities conducted by CDC researchers. MarketScan data were accessed and extracted by the first author (S.D.G.) using Merative MarketScan Treatment Pathways, an online analytic platform that includes data from participating health plans reporting outpatient pharmacy records for their enrollees. Data tabulations were prepared using Microsoft Excel (Redmond, WA, USA).

Medicaid data from CMS contain person-level, deidentified administrative claims and encounters for publicly insured children and adults. The CMS Medicaid data include records for individuals enrolled in Medicaid and Children’s Health Insurance Program (CHIP) plans that contributed data. Records for more than 25 million unique enrollees of all ages from 2017 to 2023 were accessed by the second author (K.H.) through a unique CDC–CMS data use agreement that provides access to selected CDC researchers for approved projects. The Medicaid data include records for more than half of all US births during these years. Data tabulations were prepared using Microsoft Excel.

We created calendar-year birth cohorts for both the Commercial and Medicaid databases. Identification of birth cohorts in the CMS Medicaid data was based on recorded dates of birth. Because the MarketScan research databases do not list dates of birth, we used diagnosis and procedure codes, age in years, and inpatient place-of-service codes to identify presumed birth hospitalizations in the MarketScan Commercial analysis. More than 98% of US infants are born in hospitals [22].

Administratively reported International Classification of Diseases, 10th Revisions (ICD-10) diagnosis codes for infantile SMA, type I (G12.0), other specified SMA (G12.1, G12.8), or unspecified SMA (G12.9) were used to identify potential SMA cases. Our administrative case definition of presumptive SMA diagnoses involved an algorithm requiring the presence of an SMA diagnosis code on 1 or more inpatient claims or 3 or more outpatient claims at least 6 days apart, recorded within the first 4 years of life or prior to disenrollment. Because the accuracy of diagnosis codes is higher for inpatient claims than for outpatient records, health services researchers typically require the presence of diagnosis codes in more than one outpatient claim [23]. For the same reason, previous analyses of healthcare administrative databases for SMA cases have required either 1 inpatient record or 2 or more outpatient claims or encounters with SMA diagnosis codes [24,25], 2 or more claims in any setting with SMA codes [26,27], or a minimum of 1 inpatient record or 3 or more outpatient records with SMA codes [28,29].

We assessed the administrative prevalence of SMA at specific timepoints by calculating the timing of the first SMA record for presumptive SMA cases relative to birth (Medicaid) or birth hospitalization (Commercial), recorded prior to 1, 3, 12, or 24 months. Our primary measure of timeliness of case detection was the percentage of all cases detected by the age of 24 months that had a first claim with an SMA diagnosis code recorded by 1 month of age. For the 2022 Medicaid and 2023 Commercial birth cohorts, which by definition could not have 24 months of follow-up data, we report the percentage of cases meeting the SMA case algorithm by 12 months of age that had a first SMA diagnosis code recorded by 12 months of age.

## 3. Results

### 3.1. CMS Medicaid Data

Table 1 shows the total number of presumed SMA cases by age in the Medicaid database and the corresponding numbers of live births for each birth cohort, which declined from 2.2 million in 2016 and 2017 to <2.0 million after 2019.

Table 2 shows the calculated administrative prevalence of presumed SMA cases at ages 1 to 24 months for each Medicaid birth cohort from 2016 to 2022, derived from the numbers reported in Table 1. The administrative birth prevalence (assessed at age 24 months) during 2017–2019 ranged from 6.5 to 6.9 per 100,000 births, which is similar to the roughly 7 per 100,000 birth prevalence reported in state program data [20,21]. The administrative birth prevalence was higher in the Medicaid 2020 and 2021 birth cohorts, ranging from 8.5 to 8.9 per 100,000 births.

The percentage of SMA cases recorded in Medicaid data by age 24 months that had been diagnosed by 1 month of age increased throughout the study period, from 13.5% in 2016 to 60.6% in 2021. The percentage of cases recorded by 12 months of age that had been detected by 1 month of age, a secondary measure of timing, further increased from 63.9% (101/158) in 2021 to 76.4% (133/174) in 2022.

### 3.2. MarketScan Commercial Data

Almost no SMA diagnosis codes at any age were recorded for the 2017 Commercial birth cohort (n = 3). The absolute numbers of presumed SMA cases meeting the case definition by age 48 months fluctuated in the Commercial birth cohorts from 2018 to 2021, ranging from 9 to 15 (Table 3).

Table 4 shows the administrative prevalence of SMA at ages 1, 3, 12, and 24 months for the 2018–2023 Commercial birth cohorts. The administrative prevalence of SMA in the Commercial birth cohorts by 24 months of age increased over time, from 6.1 per 100,000 in 2018 to 10.8 per 100,000 in 2023. The percentage of cases detected by 24 months of age that had SMA diagnosis codes recorded in the first month of life increased rapidly, from 9.1% in 2018 to 83.3% in 2022. In the 2023 birth cohort, 100% of infants with a diagnosis of SMA by 12 months of age had a diagnosis code recorded by 1 month, compared with 83.3% of the 2022 birth cohort using the same metric of timely detection.

## 4. Discussion

This is the first published report of nationwide trends in the detection of SMA among US infants assessed using two administrative health data sources. Our primary results are based on CMS Medicaid claims data, which comprise more than half of US births (3.9 million in 2017 and 3.7 million in 2022 [30]). In comparison, the MarketScan Commercial cohorts accounted for <5% of US births, and the precision and reliability of the estimates are subject to greater uncertainty owing to the small number of infants with SMA.

Implementation of SMA NBS in Utah was associated with detection in the first month of life for all infants with SMA born in Utah during the first 5 years of screening [16].

In the Medicaid database, timely detection (by 1 month) among cases detected by 24 months of age rose from 23.4% for the 2018 birth cohort to 60.6% for the 2022 birth cohort. Although the 2022 birth cohort did not have complete follow-up through 24 months of age, as the last records were for December 2023, the proportion of SMA cases detected by 12 months of age that had a first SMA diagnosis code recorded in the first month of life was 76.4%. In comparison, the proportion of infants in the MarketScan database with presumptive diagnoses detected by 24 months that had an SMA diagnosis code recorded by 1 month of age increased from 9.1% in the 2018 cohort to 83.3% in the 2022 cohort, all of whom potentially had follow-up data through 24 months of age. The higher percentage of SMA cases detected by 1 month in the 2022 privately insured cohort (83.3%) compared with the publicly insured cohort (60.6%) may reflect differential access, but the numbers were too small for robust inference.

Timely diagnosis of SMA supports presymptomatic initiation of disease-modifying therapies. The NURTURE study found that initiation of nusinersen between 3 and 42 days of age was associated with significantly improved outcomes for infants with 2 or 3 *SMN2* copies relative to historical controls [10,31]. Outside of clinical trials, however, newborn screening does not necessarily ensure timely initiation of treatment, which may be delayed or declined for a variety of reasons [32]. US providers report that delays in insurance approvals can delay initiation of treatment [21]. In Utah, during the first 5 years of screening, the median age at treatment was 19 days for four infants with 2 *SMN2* copies and 47 days for five infants with 3 *SMN2* copies [16]. In European countries where insurance preapproval is not required, other factors, including elevated AAV9 antibody titers in the infant and prematurity, can delay initiation of OA gene therapy [33].

Follow-up data from two German states that had implemented screening for SMA found that, of 44 infants detected with 2 or 3 *SMN2* copies, the mean age at initiation of treatment was 1.3 months, with a skewed distribution and a standard deviation of 2.2 months [32]. The same study reported that, in data from a German and Austrian clinical SMA registry, similar patients diagnosed in areas without screening began treatment at an average age of 10.7 months, with worse survival and motor function than the screened cohort. The magnitude of the reduction in age at initiation of treatment during the early phase of SMA screening implementation in Germany may be conservative if timely detection and intervention continue to increase over time.

In the Medicaid data, the percentage of infants with presumed SMA detected within 24 months of birth that had an SMA claim within the first month rose from 13.5% in 2016 to 23.4% in 2018 (Table 2). That increase preceded the widespread implementation of SMA NBS. Testing for SMA soon after birth in the absence of NBS may have been based on having an affected older sibling. Other newborns may have been tested for SMA because their mother had tested positive on carrier screening. Carrier screening for SMA has been recommended for patients who are pregnant or seeking to become pregnant by the American College of Medical Genetics and Genomics since 2008 and by the American College of Obstetrics and Gynecology since 2017 [34]. Finally, increases in early diagnosis of SMA may have reflected increased clinical awareness of SMA associated with the FDA approval of nusinersen in 2016.

The findings are subject to limitations. One limitation is attrition due to disenrollment from health plans. For example, roughly 10–15% of infants in the Medicaid birth cohorts appear to have disenrolled from Medicaid or CHIP prior to 24 months (results not reported). Furthermore, the accuracy of diagnosis codes for billing purposes is subject to uncertainty. Administrative databases contain records generated as byproducts of reporting or paying for services and do not contain patient-reported or clinical information. Diagnosis codes are recorded as reasons for healthcare encounters; the presence of a diagnosis code for a condition such as SMA indicates that the visit was related to SMA but does not necessarily mean that a clinical diagnosis had been established. Although it is possible for researchers to assess the validity of diagnosis codes or code-based case definitions through linkage of administrative and clinical databases, such studies are often not available for rare disorders, and no validation studies for SMA diagnosis codes have been published.

Although the optimal approach for identifying SMA cases in administrative data has not been established, the administrative birth prevalence estimates of SMA reported here appear generally consistent with published estimates. According to US NBS data, the birth prevalence of SMA is just under 7 per 100,000 live births [17]. On the other hand, if diagnosis codes for SMA are sometimes carried over in electronic health records without final confirmation, the number of presumed SMA cases in recent birth cohorts could be overstated by our algorithm, which treats the presence of SMA diagnosis codes in two or more outpatient claims at least one week apart as equivalent to a presumptive diagnosis.

Another limitation is that neither database includes complete records of services received for individuals with both Medicaid and private coverage. The CMS Medicaid database contains a flag variable to identify enrollees with dual coverage through other insurance types. Approximately 5% of infants with Medicaid coverage in this study also had private insurance or other third-party coverage. It is unlikely that missing records for services reimbursed by non-Medicaid payers affected estimates of administrative prevalence, since dually enrolled infants with SMA likely had encounters with SMA diagnosis codes recorded in both Medicaid and private insurance databases.

Further analyses of claims databases with information on the state of birth could assess the timeliness of detection relative to the timing of SMA NBS implementation across states. The platform used in this preliminary analysis to access MarketScan Commercial claims data does not allow for identification of the state of birth.

## 5. Conclusions

This preliminary analysis of US health insurance claims data confirms the increase in the detection of SMA in early infancy following the implementation of SMA screening in US NBS programs beginning in 2018. Timely detection by 3–4 weeks of age enables initiation of treatment before 6 weeks of age. Future analyses of the timing of treatment initiation could examine clinical registry and survey data with more complete records of treatments.

## Figures and Tables

**Table 1:** Numbers of infants with SMA diagnosis codes recorded by 1, 3, 12, and 24 months of age, and the number meeting the SMA case definition by 48 months of age, for the 2016–2022 CMS Medicaid birth cohorts.

Birth Cohort	Live Births	First SMA Code by Age 1 Month	First SMA Code by Age 3 Months	First SMA Code by Age 12 Months	First SMA Code by Age 24 Months	Total SMA Detected by Age 48 Months
2016	2,246,783	17	41	103	126	140
2017	2,197,093	28	49	125	153	188
2018	2,106,098	35	56	115	147	175
2019	2,048,986	49	84	128	142	159
2020	1,960,900	87	113	158	180	NA ^1^
2021	1,944,076	101	128	158	169	NA ^1^
2022	1,972,770	133	152	174	NA ^2^	NA ^1^

^1^ NA: Not available, as the potential length of follow-up was less than 1459 days after birth. ^2^ NA: Not available, as the potential length of follow-up was less than 730 days after birth.

**Table 2:** Administrative prevalence of SMA per 100,000 live births at 1, 3, 12, and 24 months of age for the 2016–2022 CMS Medicaid birth cohorts, and the percentage of cases detected by 24 months of age with SMA diagnosis codes recorded in the first month of life.

Birth Cohort	By 1 Month	By 3 Months	By 12 Months	By 24 Months	Percent of Those Detected by 24 Months Who Were Detected by 1 Month
2016	0.8	1.8	4.6	5.6	13.5%
2017	1.3	2.2	5.6	6.9	18.4%
2018	1.5	2.5	5.2	6.5	23.4%
2019	2.1	3.9	6.0	6.6	32.4%
2020	4.4	5.7	7.9	8.9	49.1%
2021	5.1	6.4	8.0	8.5	60.6%
2022	6.6	7.5	8.5	NA ^1^	NA ^1^

^1^ NA: Not available, as the potential length of follow-up was less than 730 days after birth for the 2022 birth cohort.

**Table 3:** Number of SMA diagnosis codes recorded by 1, 3, 12, and 24 months of age, and the number meeting the SMA case definition by 48 months of age for the 2017–2023 Merative^®^ MarketScan Commercial birth cohorts.

Birth Cohort	Live Births	First SMA Code by Age 1 Month	First SMA Code by Age 3 Months	First SMA Code by Age 12 Months	First SMA Code by Age 24 Months	Total SMA Detected by Age 48 Months
2017	200,810	0	1	2	3	3
2018	185,372	1	3	8	11	11
2019	173,079	7	9	13	15	15
2020	158,203	7	7	8	8	9
2021	150,659	8	9	10	11	11
2022	142,260	10	11	12	12	NA ^1^
2023	141,926	15	15	15	NA ^2^	NA ^1^

^1^ NA: Not available, as the potential length of follow-up was less than 1459 days after birth. ^2^ NA: Not available, as the potential length of follow-up was less than 730 days after birth.

**Table 4:** Administrative prevalence of SMA per 100,000 live births at 1, 3, 12, and 24 months of age for the 2018–2023 Merative^®^ MarketScan Commercial birth cohorts, and the percentage of cases detected by 24 months of age with SMA diagnosis codes recorded in the first month of life.

Birth Cohort	By 1 Month	By 3 Months	By 12 Months	By 24 Months	Percent of Those Detected by 24 Months Who Were Detected by 1 Month
2018	0.6	1.7	4.4	6.1	9.1%
2019	4.1	5.3	7.7	8.9	46.7%
2020	4.6	4.6	5.2	5.2	87.5%
2021	5.4	6.1	6.8	7.4	72.7%
2022	7.1	7.8	8.6	8.6	83.3%
2023	10.8	10.8	10.8	NA ^1^	NA ^1^

^1^ NA: Not available, as the potential length of follow-up was less than 730 days after birth for the 2023 birth cohort.

## Data Availability

The research data used in this study are not publicly available because they are proprietary databases licensed for use by CDC staff under approved data use agreements and cannot be shared with other individuals or groups.

## Abbreviations

The following abbreviations are used in this manuscript:

SMA	Spinal muscular atrophy
SMN	Survival motor neuron
SCID	Severe combined immunodeficiency
NBS	Newborn screening
OA	Onasemnogene abeparvovec
RUSP	Recommended Uniform Screening Panel
US	United States
CMS	Centers for Medicare & Medicaid Services
CDC	Centers for Disease Control and Prevention
CHIP	Children’s Health Insurance Programs
FDA	Food and Drug Administration
AAV9	Adeno-associated virus serotype 9
ICD	International Classification of Diseases
